# Improved *Brassica rapa* reference genome by single-molecule sequencing and chromosome conformation capture technologies

**DOI:** 10.1038/s41438-018-0071-9

**Published:** 2018-08-15

**Authors:** Lei Zhang, Xu Cai, Jian Wu, Min Liu, Stefan Grob, Feng Cheng, Jianli Liang, Chengcheng Cai, Zhiyuan Liu, Bo Liu, Fan Wang, Song Li, Fuyan Liu, Xuming Li, Lin Cheng, Wencai Yang, Mai-he Li, Ueli Grossniklaus, Hongkun Zheng, Xiaowu Wang

**Affiliations:** 1 Institute of Vegetables and Flowers, Chinese Academy of Agricultural Science, 100081 Beijing, China; 2 College of Horticulture, China Agricultural University, 100193 Beijing, China; 3 Biomarker Technologies Corporation, 101300 Beijing, China; 4 Department of Plant and Microbial Biology, University of Zürich, 8008 Zürich, Switzerland; 5 Shandong Provincial Key Laboratory of Protected Vegetable Molecular Breeding, Shandong Shouguang Vegetable Seed Industry Group Co. Ltd, 262700 Shouguang, Shandong Province China; 6 Forest Dynamics, Swiss Federal Research Institute WSL, 8903 Birmensdorf, Switzerland

**Keywords:** Comparative genomics, Genome evolution

## Abstract

*Brassica rapa* comprises several important cultivated vegetables and oil crops. Current reference genome assemblies of *Brassica rapa* are quite fragmented and not highly contiguous, thereby limiting extensive genetic and genomic analyses. Here, we report an improved assembly of the *B. rapa* genome (v3.0) using single-molecule sequencing, optical mapping, and chromosome conformation capture technologies (Hi-C). Relative to the previous reference genomes, our assembly features a contig N50 size of 1.45 Mb, representing a ~30-fold improvement. We also identified a new event that occurred in the *B. rapa* genome ~1.2 million years ago, when a long terminal repeat retrotransposon (LTR-RT) expanded. Further analysis refined the relationship of genome blocks and accurately located the centromeres in the *B. rapa* genome. The *B. rapa* genome v3.0 will serve as an important community resource for future genetic and genomic studies in *B. rapa*. This resource will facilitate breeding efforts in *B. rapa*, as well as comparative genomic analysis with other *Brassica* species.

## Genomes: a new map of field mustard

A detailed third-generation genome sequence has been published for *Brassica rapa*, which includes crops such as napa cabbage, turnips, and bok choy. Older technologies required researchers to stitch together many short sequences. Recently developed methods produce long, high-fidelity sequences. Xiaowu Wang at the Chinese Academy of Agricultural Science and the Shandong Provincial Key Laboratory of Protected Vegetable Molecular Breeding, Hongkun Zheng at Biomarker Technologies Corporation, and coworkers in China assembled the new version using 1498 sequences, compared to the 96,883 needed for the previous version. These methods provide a significant improvement in the level of detail, and permit mapping of highly repetitive regions, which were nearly impossible to map using older methods. The new sequence will aid breeding efforts, permit better comparisons with other *Brassica* genomes, and improve our understanding of genome evolution.

## Introduction

The genus *Brassica* comprises various economically important species, many of which are extensively cultivated around the world as oil crops and vegetables. The six *Brassica* species comprise the “triangle of U”^1^, which includes the three diploid species *B. rapa* (A genome), *B. nigra* (B genome), and *B. oleracea* (C genome), as well as the three amphidiploid species *B. juncea* (A and B genomes), *B. napus* (A and C genomes), and *B. carinata* (B and C genomes). The *Brassica* genomes not only underwent an additional whole-genome triplication event after divergence from *Arabidopsis thaliana*^2^ but also shared very recent genome duplications. These features make the *Brassica* genus an interesting system for the study of genome evolution in polyploids.

The *B. rapa* genome was the first to be sequenced among the *Brassica* species^2^. The first released genome draft, *B. rapa* genome v1.5, was created using a whole-genome shotgun strategy with Illumina short reads and facilitated genome assemblies of other *Brassica* species^3–5^. A more recent release, *B. rapa* genome v2.0 (ref.^6^), resulted from iterative updates with additional short read data. It was further updated to the *B. rapa* genome v2.5 after improving the scaffold order (http://brassicadb.org/brad/datasets/pub/Genomes/Brassica_rapa/V2.0/V2.5/). Since 2011, both genetic and comparative genomic studies in *Brassica* species have benefited from the *B. rapa* draft sequences. Due to the relatively recent whole genome triplication, the *B. rapa* genome harbors highly repeated sequences and complicated centromeric regions, making it difficult to assemble the genome with high accuracy using short read technologies only. The inaccuracy in assembly and the low contiguity of the current draft assemblies have limited applications in both genomic and genetic studies of *B. rapa*.

Transposable elements (TEs) play an important role in genome expansion and evolution. Based on their mechanism of transposition, TEs are categorized into class I (retrotransposons) or class II (DNA transposons) TEs^7^.Retrotransposons, especially those belonging to the long terminal repeat retrotransposon (LTR-RT) class, are the most abundant and diverse TEs in plant genomes. LTR-RTs can be primarily classified into two super families, *Ty1/Copia* and *Ty3/Gypsy*^8^. Although LTR-RTs are conserved in structure, significant variations have been observed, even among closely related *Brassica* species^4,6^. In *B. rapa*, two waves of LTR-RT expansion were identified since the divergence of *B. oleracea* and *B. rapa*^6^. However, only one wave of LTR-RT expansion was identified in *B. oleracea*^4^. Unfortunately, using the current assemblies of *B. rapa*, previous studies were unable to identify other important features of TEs. Owing to the highly repetitive nature of TEs, improving the quality of the genome assembly will allow the detection of more TEs in the *B. rapa* genome.

Comparative analysis of *B. rapa* and *A. thaliana* using ancestral genomic blocks of the Ancestral Crucifer Karyotype (ACK)^9^ identified 71 of the 72 expected genome blocks (3 × 24) in the *B. rapa* genome^10^. According to their rate of gene loss (fractionation), genome blocks were classified as belonging to the LF (the least fractionated), MF1 (the medium fractionated), and MF2 (the most fractionated) subgenomes^2^. Comparison to the ACK and alignment of centromere-specific repeats highlights the centromeric locations in the *B. rapa* genome^10^. Although the previous genome assemblies provide extensive information^2,6^, there is room for improvements in the accuracy of defining the relationships of the genome blocks and the locations of centromeres in the *B. rapa* genome.

Over the last few years, considerable progress has been made to improve the assembly of plant genomes through the use of single-molecule sequencing, optical mapping, and chromosome conformation capture technologies. Recently, several high-quality plant genomes were assembled using one or a combination of these technologies^11–14^. The use of single-molecule sequencing reads can overcome the limitations of short-read sequencing by producing long reads of tens of kilobases (kb), which span the repetitive regions in *Brassica*. Thus, the de novo assembly of a new reference genome for *B. rapa* using such novel third-generation technologies is imperative.

Here, we present a vastly improved assembly of *B. rapa* using a combination of single-molecule sequencing (PacBio), optical mapping (BioNano), and chromosome conformation capture (Hi-C) technologies. Our new assembly, *B. rapa* genome v3.0, achieves a high level of continuity and is of superior quality. We not only improved the relationship of genome blocks and provided accurate locations of the centromeres but also identified an additional LTR-RT expansion event in the *B. rapa* genome. The updated assembly can be utilized as a valuable resource for future genetic and genomic studies, as well as a new reference genome for *B. rapa*.

## Results

### Genome assembly

To guide genome assembly, we estimated the size of the *B. rapa* genome by flow cytometry using rice as a reference. We initially estimated that *B. rapa* has a genome size of 455 Mb (Supplementary Table S1). Further investigation involving calculations for the total length of the consensus map generated based on BioNano data indicated a genome size of 442.9 Mb (Supplementary Table S2). Both estimations were smaller than the previously reported size of 529^15^ or 485 Mb^2^.

We assembled the *B. rapa* genome using ~57-fold coverage of PacBio sequencing subreads (~25.88 Gb), ~456-fold coverage of BioNano data (~207.70 Gb), and ~164-fold coverage of Hi-C reads (~74.64 Gb).The resulting assembly consisted of 1476 contigs, with a contig N50 of 1.45 Mb and a total length of 351.06 Mb (Table 1). Subsequently, we detected discrepancies within 22 contigs using the Hi-C reads (Supplementary Table S4). Instead of removing these contigs, we split these at the conflict regions; the data for Contig01464 are shown as an example (Supplementary Figure S1).

**Table 1:** Summary of comparisons of assembly and annotation for the three *B. rapa* genome assemblies

Assembly			
	v3.0	v2.5	v1.5
Sequence genome size (Mb)	353.14^a^	389.19	283.81
GC content (%)	36.83	36.17	35.26
Number of contigs	1498	96,883	51,647
Contig N50 size (kb)	1446	53	46
Number of scaffolds	1301	86,986	40,576
Scaffold N50 size (kb)	4437	3378	1847
Gaps total number	396	10,158	11,426
Gaps total length (kb)	2078	22,776	10,710
Gaps number per Mb	1.12	25.98	40.09
Gap length (kb) per Mb	5.89	60.53	41.25
**Annotation**			
Total gene models	45,985	48,826	41,020
Tandem arrays	2077	3535	2077
Tandem genes	4963	8002	5004
Redundancy removed	43,099	44,359	38,093
Syntenic genes	39,858	40,442	35,464
Nonsyntenic genes	3241	3917	2629

^a^See Supplementary Table S3

After scaffolding and estimating gap sizes using BioNano maps and mate-pair reads (from BRAD, http://brassicadb.org), we obtained 1301 scaffolds with a scaffold N50 of 4.44 Mb (Table 1). To assign the resulting scaffolds to their chromosomal positions, we anchored these scaffolds using the Hi-C data and the improved genetic map (see Methods). We anchored 298.19 Mb of sequence on ten chromosomes that included 200 scaffolds clustered by Hi-C data and 8 scaffolds assigned by the genetic map. Our final assembly, termed *B. rapa* genome v3.0, totaled 353.14 Mb of sequence with 396 gaps (2.08 Mb) (Table 1). The *B. rapa* genome v3.0 is longer than v1.5 but shorter than v2.5.

To assess the quality of the *B. rapa* genome v3.0, we used various data sources. First, we validated the completeness of our assembly by searching for core eukaryotic genes (CEGs) using CEGMA^16^. A total of 247 out of 248 CEGs were complete, and 1 CEG was partial, indicating that all of the CEGs could be detected in our assembly (Supplementary Table S6). Next, the genome quality was tested by matching the sequences of expressed sequence tags (ESTs) of *B. rapa* (downloaded from dbEST at NCBI), which showed that 99.34% of the ESTs could be found in the newly assembled *B. rapa* genome v3.0.

### Contiguity improvement

The *B. rapa* genome v3.0 has improved contiguity in terms of gaps and contig sizes. The *B. rapa* genome v1.5 was generated from Illumina sequences, whereas more Illumina reads and a relatively small amount of PacBio sequence data were used for assembly v2.5. These two assemblies have limitations due to their fragmentation and low contiguity (Table 1). By combining single-molecule sequencing, optical mapping, and Hi-C technology, *B. rapa* genome v3.0 represents a ~27-fold (contig N50: 1446 Kb vs. 53 Kb, v2.5) and ~31-fold (contig N50: 1446 Kb vs. 46 Kb, v1.5) improvement in contiguity over the two previous assemblies (Table 1).We also assessed the size and quantity of gaps in each respective assembly. There were only 396 gaps in v3.0, including gaps of known (122 from BioNano and 74 from mate-pair data) and unknown sizes (190 from Hi-C scaffold joining and 10 from genetic map joining). Compared to the previous assemblies, v3.0 has ~10-fold (5.89 Kb vs. 60.59 Kb, v2.5) and ~7-fold (5.89 Kb vs. 40.09 Kb, v1.5) improvement in the size of gaps per Mb over the two previous assemblies (Table 1). In terms of the number of gaps per Mb, v3.0 is superior to v2.5 and v1.5, respectively, with ~23-fold (1.15 vs. 25.98, v2.5) and ~35-fold (1.15 vs. 40.09, v1.5) fewer gaps per Mb (Table 1).

**Fig. 1:**
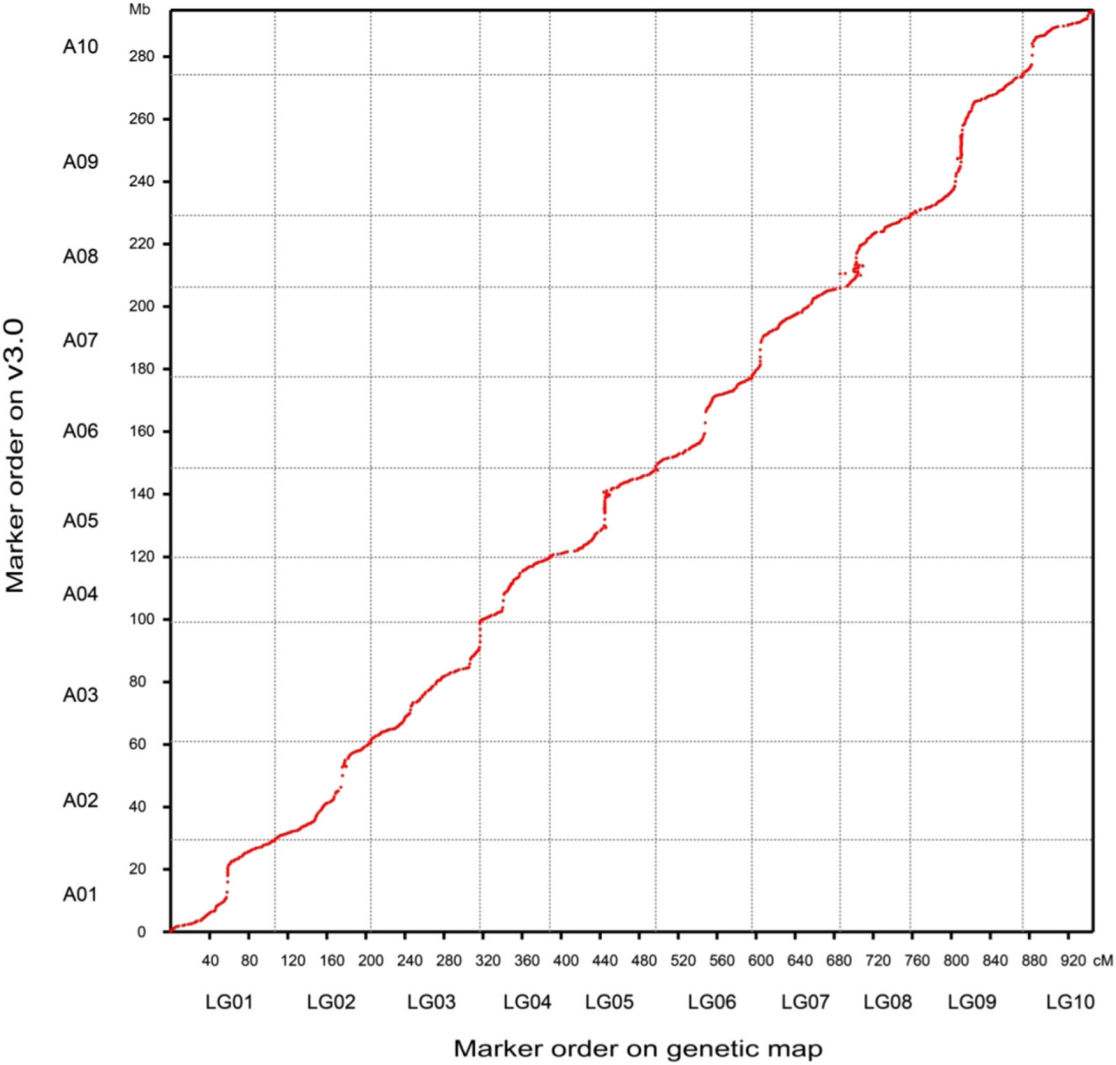
Integration of the physical and genetic maps of *B. rapa* genome v3.0. The markers of the genetic map based on *B. rapa* genome v3.0 are shown on the *x*-axis; the markers of the physical map of *B. rapa* genome v3.0 are shown on the *y*-axis

To assess the contiguity and accuracy of scaffold ordering of the three versions of the *B. rapa* reference genome, we first reconstructed the genetic maps based on the three assemblies using the same set of resequencing data of a doubled haploid (DH) population derived from a cross of two heading Chinese cabbage lines^17^. We then assessed the locations of binmarkers on the genetic maps by integrating them with the corresponding physical maps. Of the 892 binmarkers in our assembly, 877 binmarkers (98.3%) were mapped in the genetic map. Our assembly agreed with the genetic map for 801 binmarkers (91.3%), indicating the high quality of v3.0 (Fig.1; Supplementary Table S7). However, we noticed that 76 (8.7%) binmarkers on chromosomes A05, A08, and A09 mapped to ambiguous locations in the genetic map. These regions contained repeated sequences, especially at centromeric regions, as described in the following analysis. However, these conflicting regions were covered by PacBio reads and/or BioNano maps; the data for chromosome A08 in v3.0 are shown as an example (Supplementary Figure S2).

There were 1092 binmarkers on the genetic map of v2.5 and 866 binmarkers on the genetic map of v1.5. However, we could only map 88.7% of binmarkers (969 of 1092) and 92.3% of binmarkers (799 of 866) onto the genetic map of v2.5 and v1.5,respectively (Supplementary Table S7). We found that 15.1% of the binmarkers (166 of 969) in v2.5 were discrepant, including 146 binmarkers with disordered genetic and physical distances within the same chromosome (intrachromosome) and 20 binmarkers with inconsistent genetic and physical distances on different chromosomes (interchromosome) (Supplementary Figure S3; Supplementary Table S7). For v1.5, 10.0% of binmarkers (80 of 799) were discrepant, including 71 binmarkers of intrachromosome and 9 binmarkers of interchromosome (Supplementary Figure S4; Supplementary Table S7). However, v3.0 contained the least conflicting intrachromosomal binmarkers (8.7%, 76 of 877 binmarkers) and no discrepant interchromosomal binmarkers (Supplementary Table S7), indicating that *B. rapa* genome v3.0 has a higher contiguity than the two previous assemblies. Taken together, these independent validations suggest that *B. rapa* genome v3.0 has the highest contiguity and the best ordering of scaffolds among the three *B. rapa* assemblies.

### Comparison of genome annotation

We predicted and annotated the gene models as previously described^6^. We identified a total of 45,985 protein-coding gene models in v3.0, which represented 14.74% of the genome assembly (Table 1). In our assembly, 98.75% (45,411 of 45,985) of the genes were annotated on chromosomes, and only 1.25% (574 of 45,985) was located on scaffolds. The de novo annotated genes in v3.0 were named following the standard of gene model nomenclature for the *Brassica* reference genomes (http://www.brassica.info/info/genome_annotation.php). The number of gene models in the novel assembly is higher than that in v1.5 (41,020 genes) but lower than that in v2.5 (48,826 genes) (Table 1). To further evaluate the quality of the annotation, a comparison with the annotation of previous assemblies was performed using BUSCO^18^, which is based on a benchmark of 1440 conserved plant genes. Approximately 97.7% of these conserved plant genes were identified, and 1.7% were detected as fragments presented in v3.0 (Supplementary TableS11).

**Fig. 2:**
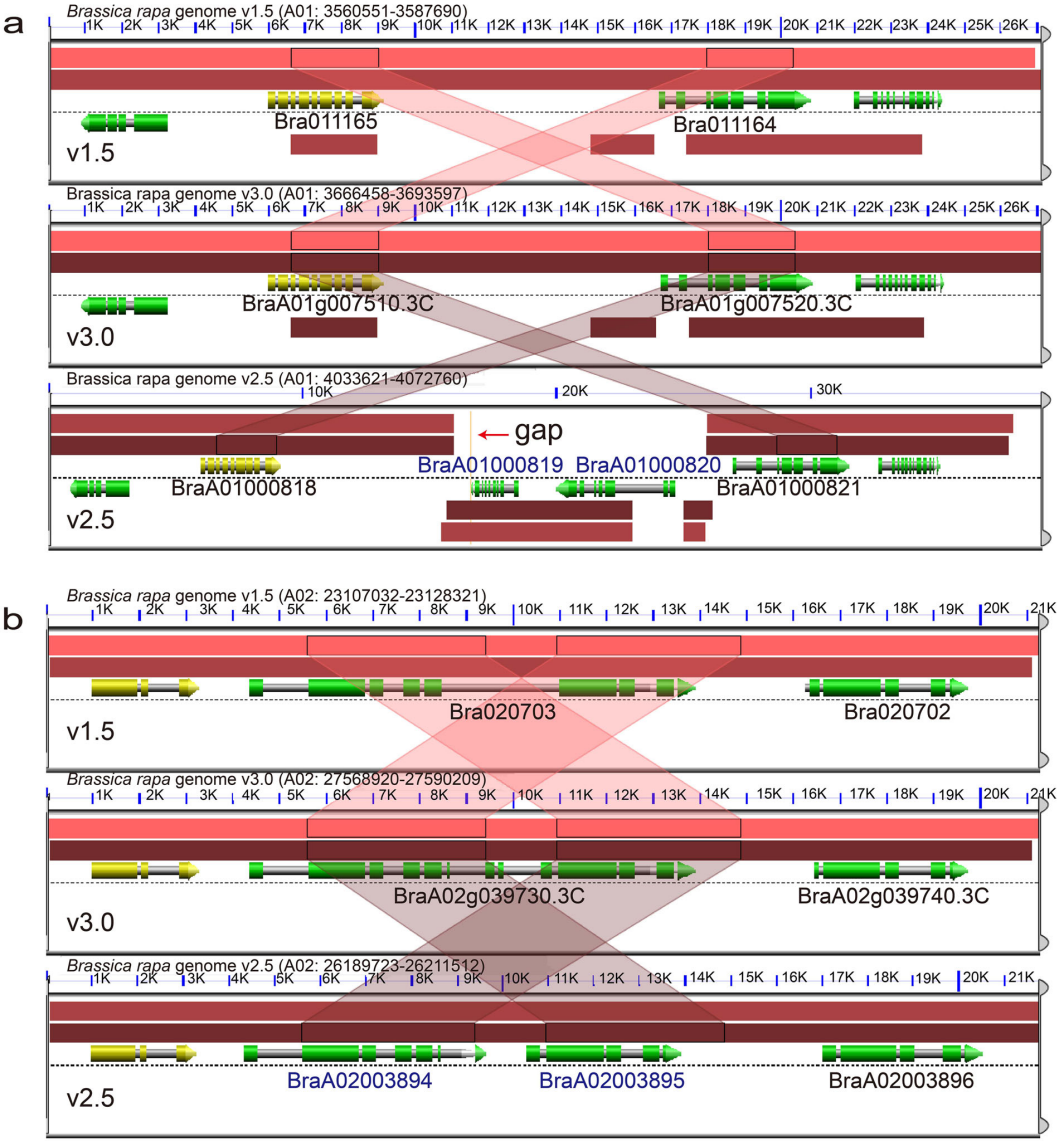
Examples showing the invalid annotation of tandem genes in v2.5. **a** An example of a 25 bp gap (thin yellow bar indicated by the red arrow) between the genes *BraA01000818* and *BraA01000819*, indicating an invalid annotation in v2.5. **b** The genes *BraA02003894* and *BraA02003895* in v2.5 are annotated as a single gene in v3.0 (*BraA02g039730.3C*) and v1.5 (*Bra020703*). Figures were plotted using GEvo (https://genomevolution.org/coge/GEvo.pl)

A genome synteny analysis was performed among the three assemblies using SynOrths^19^ to identify syntenic gene pairs and tandem gene arrays. A total of 2077 tandem arrays (corresponding to 4963 tandem genes) were identified in v3.0. The same number of tandem arrays (2077 arrays corresponding to 5004 genes) was also detected in v1.5. An assessment of genome-wide synteny indicated that 1539 tandem arrays (corresponding to 3757 genes) in v3.0 were syntenic to 1494 tandem arrays (corresponding to 3670 genes) in v1.5. However, more tandem arrays (3535 arrays, 8002 genes) were identified in v2.5 (Table 1). We detected gaps in the regions of superfluous tandem genes in v2.5, whereas no gaps were found in either 3.0 or v1.5 (Fig. 2a). These gaps may be the result of assembly errors produced by gap closing using PacBio reads in v2.5, which in turn led to the invalid annotation of tandem genes. For other tandem genes without gaps, we observed that single genes in v3.0 and v1.5 were annotated as two or more genes in v2.5 (Fig. 2b).

When taking each tandem array as a single gene locus, there were 43,099 genes remaining in v3.0, 44,359 genes in v2.5, and 38,093 genes in v1.5 (Table 1). We then performed a gene synteny analysis, which revealed that 39,858 genes (92.48%) in v3.0 served as counterparts to 40,442 (91.17%) and 35,464 genes (93.10%) in v2.5 and v1.5, respectively. After comparison of the annotated genes with those of the early versions, we identified 3241 version-specific genes in v3.0 compared to both v2.5 and v1.5. Of these, 2380 genes were supported by evidence from matching mRNA reads of *B. rapa* (from BRAD, http://brassicadb.org/), and 2295 genes were supported by protein sequences of other Brassicaceae species (Supplementary Table S12). In total, 89.10% (2888 of 3214) of the version-specific genes in v3.0 were supported by the mRNA data of *B. rapa* or the protein sequences of other Brassicaceae species, while only 10.90% (326 of 3214) of the genes were not supported.

### A new LTR-RT expansion event identified in the updated assembly

We annotated TEs in v3.0 using the same methods as previously reported^20^. A total of 235,683 TEs were identified from 1244 families in v3.0, and 562 unique TE families were found compared to v2.5 and v1.5. In v3.0, TEs representing 37.51% (134 Mb) of the assembled genome, which was higher than in the previous assemblies (32.30%, 126 Mb, v2.5; 25.44%, 72 Mb, v1.5)^2,6^. In our novel assembly, the most abundant TEs are LTR-RT, which covers a total length of 57.64 Mb and represents 16.32% of the assembled genome. Non-LTR-RT repeats (LINEs and SINEs) account for 3.10% of our assembly (Supplementary Figure S5). We detected DNA transposons corresponding to 26.35 Mb, which make up 7.46% of the assembled genome assembly (Supplementary Figure S5). A complete list of identified TEs and repeats in v3.0 can be found in Supplementary Table S13. In addition, we identified a total of 1231 miRNAs, 1281 tRNAs, 2865 rRNAs, and 3737 snRNAs in the *B. rapa* genome v3.0 (Supplementary Table S19).

**Fig. 3:**
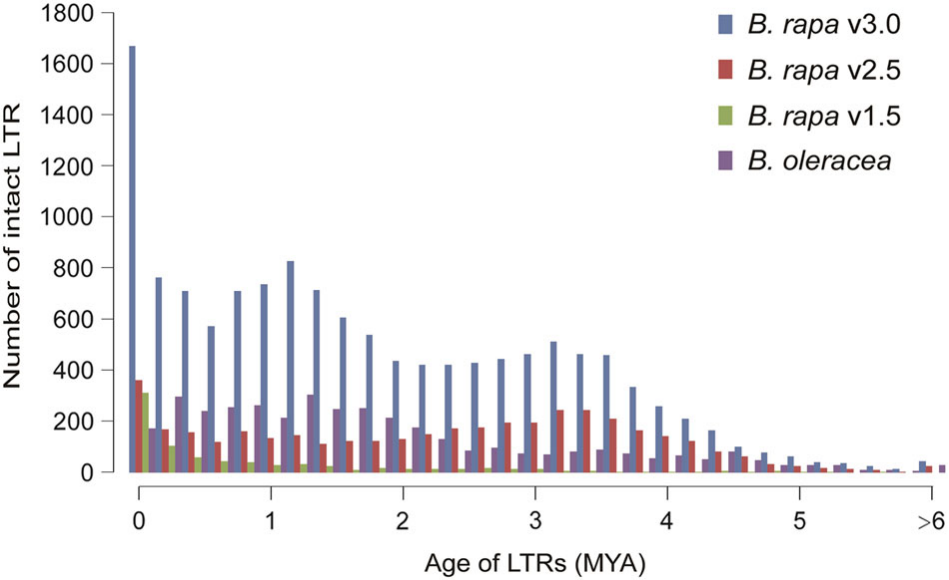
**The number of intact LTR-RTs birthed at different times (million years ago, MYA) in the three assemblies of the*B***. ***rapa*** **genome and in the genome of*B. oleracea***.

In our current assembly, we annotated more LTR-RTs (57 Mb) compared to v2.5 (44 Mb) and v1.5 (18 Mb). We identified 51,062 nonintact LTR-RTs in v3.0. Further analysis revealed that 65.27% (33,672 of 51,602) of nonintact LTRs were located on the ten chromosomes, whereas 34.73% (17,922 of 51,602) of nonintact LTR-RTs were found on the unanchored scaffolds. Using the same method^6^, a total of 13,318 intact LTR-RTs were annotated in v3.0. However, there were only 4129 and 801 intact LTR-RTs in v2.5 and v1.5, respectively^6^. Further analysis revealed that only 18.19% of intact LTR-RTs (2423 of 13,318) were located on the ten chromosomes, whereas most (81.81%, 10,895 of 13,318) intact LTR-RTs were found on the unanchored scaffolds in v3.0.The insertion time of intact LTR-RTs was calculated as previously described^4^, which indicated that the *B. rapa* genome underwent three waves of LTR-RT expansion since it diverged from *B. oleracea* (Fig. 3). These intact LTR-RTs had an average insertion age of 1.88 million years ago (MYA), with a median insertion age of 1.59 MYA. Furthermore, we found more intact LTR-RTs with different lengths in v3.0 compared to in v2.5 and v1.5 (Supplementary Figure S6).

With these intact LTR-RTs, a new LTR-RT expansion event was identified in the *B. rapa* genome. We designated 3155 intact LTR-RT insertion events from 0 MYA to 0.4 MYA as a “young expansion” with an average length of 8135 bp and an average insertion date of 0.2 MYA; 2283 intact LTR-RT insertion events from 1.0 MYA to 1.4 MYA as a “medium expansion” with an average length of 11,902 bp and an average insertion date of 1.2 MYA; and 1444 intact LTR-RT insertion events from 3.0 MYA to 3.4 MYA as an “ancient expansion” with an average length of 9823 bp and insertion date (Fig. 3).The young and ancient expansions correspond closely to the previously identified expansion events[6]; the medium expansion was first identified in the *B. rapa* genome and has a similar insertion time as that of the intact LTR-RT expansion event in *B. oleracea*[4]. Furthermore, 1778 Ty1/Copia-like LTR-RTs and 4179 Ty3/Gypsy-like LTR-RTs were identified in v3.0, which is much more than those identified in the previous assemblies (353 Ty1/Copia and 632 Ty3/Gypsy in v2.5, 260 Ty1/Copia and 162 Ty3/Gypsy in v1.5) (Supplementary Table S20; Supplementary Figure S7, S8). In general, there were more Ty3/Gypsy-like LTR-RTs than Ty1/Copia-like LTR-RTs (Supplementary Table S20). Compared to v2.5 and v1.5, Ty3/Gypsy-like LTR-RTs in v3.0 were obviously increased since 5 MYA (Supplementary Figure S7), while Ty1/Copia-like LTR-RTs were increased since 2.2 MYA (Supplementary Figure S8). From the phylogenetic trees, we found that each group of LTR-RTs had more copies in v3.0 than in v2.5 and v1.5 (Supplementary Table S21, S22; Supplementary Figure S9, S10).

### Genome blocks and centromeres in the *B. rapa* genome

**Fig. 4:**
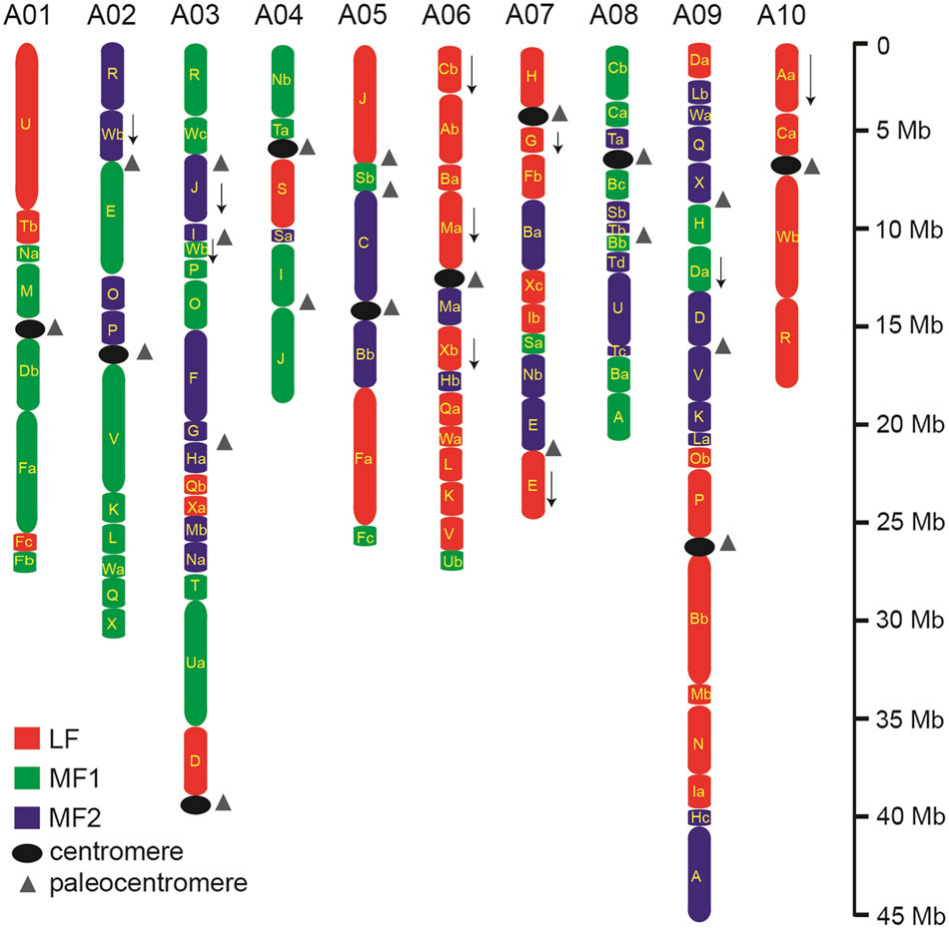
Distribution of genomic blocks along ten chromosomes of the *B. rapa* genome v3.0. Genome blocks on ten chromosomes were assigned to the subgenomes LF (red), MF1 (green), and MF2 (blue). Two or more segments of a single block were labeled using lowercase letters (a, b, etc.). The centromeres in the *B. rapa* genome are shown as black ovals, and the paleocentromeres are shown as gray triangles. Downward-pointing arrows are adjacent to GBs that are inverted relative to other blocks that originated from a single ACK chromosome

We investigated the relationships of genome blocks using the updated assembly v3.0. To define the genome blocks and centromeres in the *B. rapa* genome v3.0, we first constructed the three subgenomes (LF, MF1, and MF2) based on the syntenic relationship between v3.0 and *A. thaliana* (Supplementary Figure S11; Supplementary Table S14). We detected 71 out of the 72 (3 × 24) expected genomic blocks in v3.0, and most of them were arranged in line with those previously reported in ref.^10^ (Fig. 4; Supplementary Table S15). In v3.0, the two new fragmented genome blocks F (LF) and F (MF1) were identified on chromosomes A01 and A05 and were not observed in ref.^10^. We could not detect two previously described, very small genome blocks in v3.0, block C (MF2) on chromosome A07 and block B (MF1) on chromosome A08 (ref.^10^). However, in our assembly, genome blocks N/M (MF1), O/P (LF), and A/C(LF) were arranged on chromosomes A01, A09, and A10, respectively, whereas they were ordered on opposite sides in ref.^10^.The three small adjacent genome blocks(S (MF2), T(MF2), and B(MF1)) on chromosome A08 of v3.0 were ordered S/T/B, whereas these were arranged as T/B/S in ref.^10^.

We also compared the orientation of genome blocks in v3.0 with that in ref.^10^. The genome blocks W (MF2) on chromosome A02, as well as G (LF) and E (LF) on chromosome A07, were found to be inverted relative to the other blocks that originated from a single ACK chromosome. However, the orientation of genome block P (LF) on chromosome A09 and three blocks of V in v3.0 were in the forward direction, whereas these were inverted in ref.^10^. These results were further supported by the genetic maps of v3.0 and v1.5, respectively.

We accurately determined the location of the centromeres of all chromosomes in v3.0. By screening previously determined centromeric repeat sequences, including centromeric satellite repeats CentBr, CRB, TR238, and PCRBr^21–23^, we identified the signals for all 21 paleocentromericregions in v3.0, whereas three paleocentromeric regions were not detected in ref.^10^ (Fig. 4, Supplementary Table S16). Paleocentromere analysis indicated that the ten extant *B. rapa* centromeres were all inherited from the 21 paleocentromeres. In v3.0, the centromeres of chromosomes A01, A03, A04, A05, A06, A07, and A10 had the same associated genome blocks flanking the corresponding centromeres as reported in ref.^10^ (Fig. 4). However, the centromere on chromosome A02 was located between genome blocks P (MF2) and V (MF1), and the centromere on chromosome A09 was situated between genome blocks P (LF) and B (LF), whereas these were deemed paleocentromeres in ref.^10^ (Fig. 4). The centromere on chromosome A08 was located between genome blocks T (MF2) and B (MF1), rather than between genome blocks C (MF1) and T (MF2), as reported in ref.^10^. Furthermore, there were 1188 genes detected within centromeric regions in v3.0, whereas only 740 genes were detected in ref.^10^ (Supplementary Table S17).

**Fig. 5:**
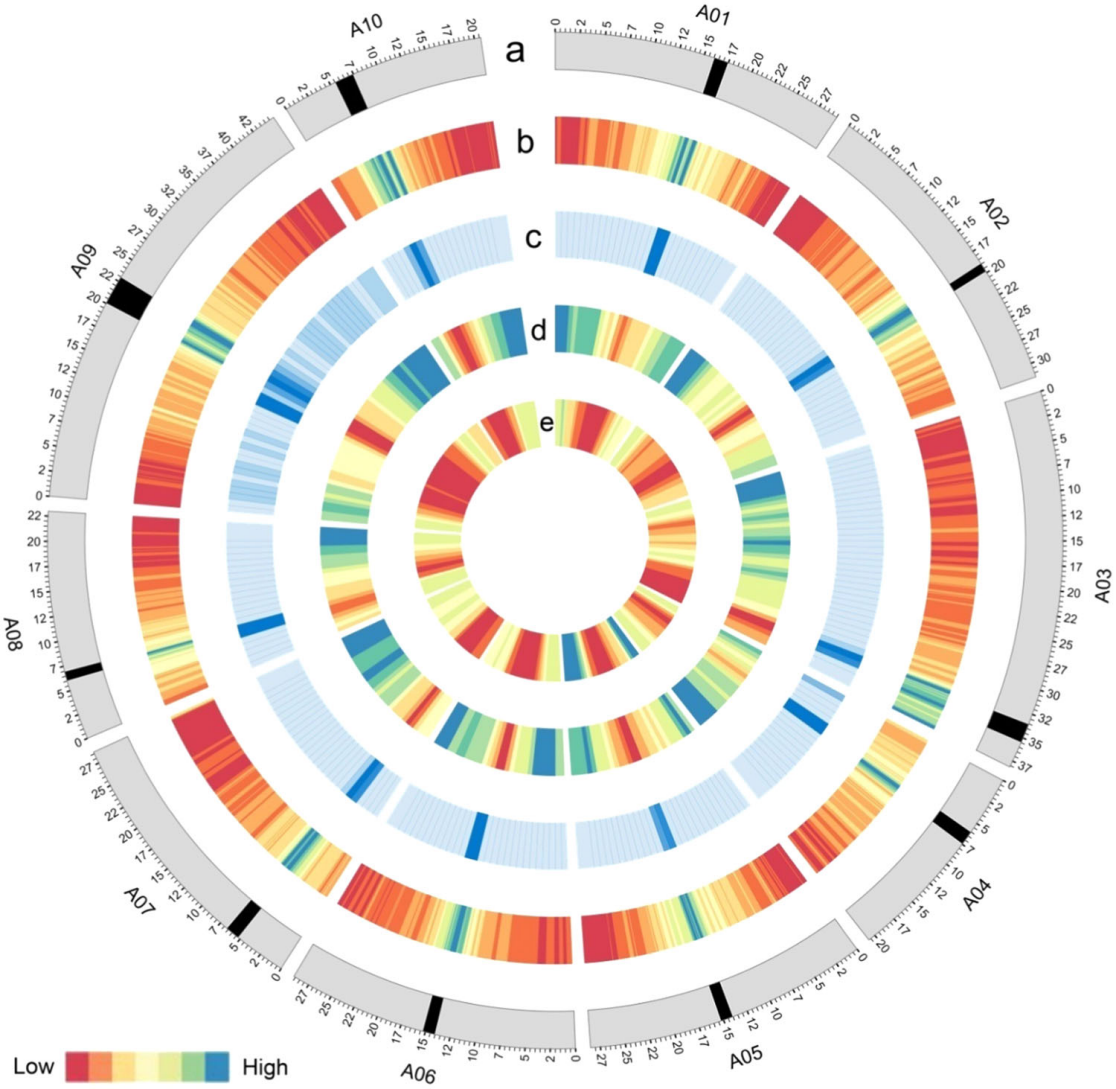
Circos plot of the features of centromeric regions on the ten chromosomes in *B. rapa* genome v3.0. All the data are represented as heatmaps. The red color indicates low values, and the blue color indicates high values. **a** The ten chromosomes of the *B. rapa* genome v3.0. Centromeres are shown as black blocks. **b** TE density across the ten chromosomes of v3.0 (500 kb sliding window, 100 kb step). **c** Distribution of centromere-specific repeats along the ten chromosomes of v3.0 (2 Mb sliding window, 1 Mb step). **d** Gene density of the ten chromosomes of v3.0 (2 Mb sliding window, 1 Mb step). **e** The mean local recombination rate between markers along the ten chromosomes of v3.0 (5 Mb sliding window, 1 Mb step)

To assess our assembly with regard to the centromeres in v3.0, we analyzed the sequence features of the centromeric regions. We found that a significantly higher number of TEs and centromere-specific repeats were mapped to the centromeric regions than to other parts of the chromosomes, and the gene density and recombination rate were markedly lower at the centromeric regions annotated in v3.0 (Fig. 5). In addition, more centromere-specific repeats were detected at the centromeric regions in v3.0 in comparison to those reported in ref.^10^ (Supplementary Table S17).

## Discussion

We present the first long-read genome assembly of *B. rapa*. The advent of PacBio sequencing resulted in a dramatically improved contig N50 size compared to that of previous assemblies (Table 1). We note that PacBio reads tend to provide excellent results to fill gaps in assemblies stemming from short reads, as demonstrated by our current assembly and other studies^14,24^. Optical mapping could not be performed using entire chromosomes due to the insufficiency of long-range spanning fragments^5^. Scaffolding with Hi-C facilitates the accurate assignment to chromosomal positions, as supported by our data on genetic markers.

Using the high-density genetic map derived from cross *B. rapa* lines with the same morphotype, we could accurately compare the quality of v3.0 with that of previous assemblies, indicating that our novel assembly achieved the highest contiguity and quality among the three assemblies. In previous assemblies, genetic maps derived from crosses between distantly related cultivars of *B. rapa* were used to assign scaffolds to chromosomes, which may have resulted in errors in the assembly. Although the length of v2.5 after the removal of “N”s was still longer than that of v3.0, it is possible that there were overlapping sequences in v2.5. In our assembly, most of the scaffolds were anchored by Hi-C data on chromosomes, and the remaining scaffolds were assigned using the genetic map. Although there were a few scaffolds that were syntenic to *A. thaliana*, we could not find any support from either Hi-C scaffolding or the genetic map. Further analysis suggested that there were more repeat sequences in these unanchored scaffolds, as we found fewer genes and more intact LTR-RTs in these scaffolds compared to those on chromosomes in v3.0. To alleviate this problem in the future, the length of input contigs using more PacBio sequencing reads could be increased or a restriction enzyme with a higher frequency of recognition sites for Hi-C scaffolding could be selected.

Our newly de novo annotated gene models were fewer than reported in v2.5. After removing the redundant tandem duplicated genes in each assembly, the number of gene models in v3.0 was closer in number to that in v2.5. A factor contributing to the observed decrease in gene number might be the overestimation of tandem duplicated genes in v2.5 due to the fragmentation of the genome assembly, which led to the annotation of sections of genes located on different contigs (Fig. 2).The gene model nomenclature in v3.0 followed the standard for reference *Brassica* genomes (http://www.brassica.info/info/genome_annotation.php), making it possible to distinguish the genes in v3.0 from those of other lines of *B. rapa*.

As hotspots for TE insertions, centromeric regions are highly repetitive and have a relatively low gene density compared to the other parts of chromosomes. Thus, locating centromeric regions on a genome sequence is challenging, especially when using short reads as in the previous assemblies of the *B. rapa* genome. However, single-molecule sequencing substantially improved the assembly of repeats in v3.0, which allowed us to accurately define the locations of centromeres. Additionally, we annotated more genes within centromeric regions than in previous assemblies. These results not only enable us to investigate genes situated within these centromeric regions but also provide the basis for a functional analysis of centromeres in *Brassica*.

The ancestral genomic blocks along the ten chromosomes of *B. rapa* were previously reported by comparing the collinearity between the three subgenomes of *B. rapa* and the *A. thaliana* genome^10^. Although the previous assembly^2^ provided much information on genome blocks, some blocks and block orientations were missing or reported as being inverted in ref.^10^, which was rectified in the new assembly (Fig. 4). The refinement of the genome block arrangement in *B. rapa* will improve the resolution of interspecies comparisons of genome collinearity across *Brassicaceae*.

With the obvious improvement in contiguity in v3.0, a higher number of TEs were annotated than in previous assemblies of the *B. rapa* genome, particularly intact LTR-RTs. Further analysis identified a new LTR-RT expansion event in the *B. rapa* genome, which indicates differences in the features of LTR-RT amplification events between *B. rapa* and *B. oleracea*. Moreover, more intact LTR-RTs might be detected and additional TE features may be identified in other plant genomes via long-read sequencing in the future.

Overall, our improved assembly, *B. rapa* genome v3.0, offers unprecedented insights into genome evolution and provides novel information relevant for comparative genome studies involving *B. rapa*. Finally, the *B. rapa* genome v3.0 provides a solid foundation for future studies, not only in *B. rapa* but also in other *Brassica* species.

## Materials and methods

### De novo genome assembly

To estimate genome size, six biological replicates were analyzed by flow cytometry of *B. rapa* (accession Chiifu-401-42) using rice (*O. sativa* ssp. *japonica* cv. Nipponbare)^25^ as an internal reference (Supplementary Table S1). The genome size of an unknown ecotype of *B. rapa* was estimated at 529 Mb by flow cytometry without control analysis^15^ and at 485 Mb for Chinese cabbage (accession Chiifu-401-42) using 17-mer analysis^2^. The estimated size of the *B. rapa* genome is very close to that of the BioNano consensus map, suggesting that previous studies may have overestimated its size.

The same *B. rapa* L. ssp. *pekinensis* in bred line (Chiifu-401-42) used for the earlier assemblies v1.5 and v2.5 was used for whole-genome sequencing in this study. High-quality genomic DNA from 500 mg of frozen leaf tissues was used to generate the PacBio libraries with an insert size of 20 Kb. The libraries were then sequenced in four Sequel cells (Pacific Biosciences, CA, USA). Approximately 19.40 Gb of newly generated data and another 6.5 Gb of previous PacBio data^6^ were incorporated into our genome assembly. Next, the PacBio subreads were de novo assembled using Canu (v1.5)^26^ with default parameters. The Illumina reads obtained from BRAD (http://brassicadb.org) were mapped to the PacBio contigs using BWA (v0.7.15)^27^. This alignment was then used to polish and correct the assembly by Pilon (v1.22)^28^.

The Hi-C libraries of *B. rapa* were constructed following the procedures described in a previous study with minor modifications^29^.The resulting libraries were submitted to an Illumina HiSeq 4000 sequencing device with 2 × 125 bp reads. Overall, we obtained ~584 million usable paired-end reads from two biological replicates. After alignment, ~27.87% of these read pairs could be uniquely mapped to the initial contigs.

The optical mapping (BioNano) data were generated using the BioNano Genomics Irys system (BioNano Genomics, CA, USA). The high-molecular-weight DNA was labeled by a specific nicking enzyme *Nt.BspQ1* (New England Biolabs, MA, USA) using the IrysPrep Reagent Kit (BioNano Genomics, CA, USA) as described by the manufacturer. Molecules were then filtered by a minimum length of 100 kb and a signal-to-noise ratio of 3.5. The filtered molecules were de novo assembled into a consensus physical genome map using the BNG IrysView analysis software package using manufacturer-recommended parameters for *B. rapa* (molecular length threshold: 100 kb; minimum label per molecule: 8; maximum backbone intensity: 0.6; false positive density/100 kb: 1.5; false negative rate: 0.15%; scaling SD: 0; site SD: 0.2 kb; relative SD: 0.03; initial assembly *p* value cutoff: 1e-8; extension and refinement *p* value cutoff: 1e-9; and merge *p* value cutoff: 1e-12; autonoise adjustment and 4 iterations of computation).

### Conflict resolution

To detect conflicts in the resulting PacBio contigs, we first aligned the Hi-C reads to these contigs and assembled by Lachesis^30^. Contact maps for all contigs produced by Lachesis were drawn using the ggplot2 package (http://ggplot2.org/). We then checked the interaction signals for each sequence with the others. The detailed region in one contig was split when it had a strong signal with distant sequences (Supplementary Figure S1). We could not find conflicts using BioNano maps because of the relatively short length of the contigs in our assembly. Once all the conflicts were resolved, the corrected PacBio contigs were used for scaffolding using the BioNano maps and mate-pair reads. Finally, the resulting scaffolds were used as input for scaffolding by Lachesis^30^ with parameters “CLUSTER_N = 10, CLUSTER_MIN_RE_SITES = 31, CLUSTER_MAX_LINK_DENSITY = 2, CLUSTER_NONINFORMATIVE_RATIO = 2, ORDER_MIN_N_RES_IN_TRUNK = 21,ORDER_MIN_RES_IN_SHREDS = 19”.

### Construction of the genetic maps

To construct the genetic map of our assembly, raw data of a DH population derived from a cross of two Chinese cabbage lines^17^ were aligned to the assembled genome using BWA (v0.7.15)^27^. Only the uniquely mapped reads were used to call SNPs by SAMtools (v0.1.19)^31^. Recombination bins were constructed as previously described^32^ used as genetic markers and imported into JoinMap 4.0. Based on the locations of binmarkers, the genetic map was integrated with the physical map. To visualize the data, we plotted the genetic distance against the physical distance of the binmarkers for each chromosome. The same method was used to construct the genetic maps of v2.5 and v1.5.

### Genome annotation

We named the newly annotated gene models following the standards of gene model nomenclature for *Brassica* reference genomes (http://www.brassica.info/info/genome_annotation.php): Bra (for *Brassica rapa*) followed by the chromosome number and letter “g” (for gene). Genes from the top to the bottom of chromosomes were assigned numbers (in steps of 10) with five digits with leading zero integers. To distinguish the genes in v3.0 from the other lines of *B. rapa*, the number “3” (for the third version of *B. rapa* reference genome) and a single capital letter “C” (for variety Chiifu-401-42) were assigned after a “.” following the gene numbers; for example, BraA05g036760.3C.

After gene prediction, gene functions were assigned according to the best match of the alignments against various protein databases using BLAST v2.2.31 (E-value = 1e-5), including the KEGG^33^, Swiss-Prot, and TrEMBL databases^34^. GO terms for each gene were obtained from the corresponding InterPro entries^35^. Overall, we inferred 44,539 (96.86%) genes that were annotated based on the results from searching the protein databases (Supplementary Table S18).

Intact LTR-RTs were identified using LTR_finder^36^ and classified the intact LTR-RTs by predicting the RT domains using the Pfam database (version 26.0) and HMMER software^37^. Muscle^38^ was then employed to perform multiple RT sequence alignments, and RAxML^39^ was adopted to construct maximum likelihood (ML) trees based on the sequence alignments with 500 bootstrap replications. Finally, the interactive tree of life (iTOL)^40^ was used to plot the ML trees. The analysis of LTR insertion time was performed as previously reported^4^.

We also performed noncoding RNA annotation for our assembly. tRNA annotation was conducted using tRNAscan-SE (v1.3.1)^41^ according to its structural characteristics. Homology-based rRNAs were localized by mapping known full-length plant rRNAs to the *B. rapa* genome v3.0. snRNAs were predicted by Infenal (v1.1)^42^ using the Rfam database^43^. miRNA annotation was performed as previously described^44^.

### Genome blocks and centromere detection in the *B. rapa* genome

We first constructed the three subgenomes (LF, MF1, and MF2) following the methods^45^. Then, we defined the genomic blocks in v3.0 based on the syntenic relationship of the *B. rapa* and *A. thaliana* genomes. Centromeric repeat sequences, including those of CentBr, CRB, TR238, and PCRBr^21–23^, were aligned to v3.0 using Nucmer^46^. The signals of the centromeric repeat sequences were used as evidence supporting the localization of the centromeres (Supplementary Table S16).

### Data availability

The data sets generated and analyzed during the current study are freely available through BRAD website (http://brassicadb.org/brad/datasets/pub/BrassicaceaeGenome/Brassica_rapa/V3.0/) or in the Genome Warehouse database under accession number GWHAAES00000000 (http://bigd.big.ac.cn/gwh). All other data generated or analyzed during this study are included in this published article and its supplementary information files.

## Electronic supplementary material

Supplementary_Figures

Supplementary_Tables

Suplementary_Tables_S4

Suplementary_Tables_S5

Suplementary_Tables_S8

Suplementary_Tables_S9

Suplementary_Tables_S10

Suplementary_Tables_S14

Suplementary_Tables_S15

Suplementary_Tables_S16
