# Author Correction: Improved *Brassica rapa* reference genome by single-molecule sequencing and chromosome conformation capture technologies

**DOI:** 10.1038/s41438-019-0210-y

**Published:** 2019-11-13

**Authors:** Lei Zhang, Xu Cai, Jian Wu, Min Liu, Stefan Grob, Feng Cheng, Jianli Liang, Chengcheng Cai, Zhiyuan Liu, Bo Liu, Fan Wang, Song Li, Fuyan Liu, Xuming Li, Lin Cheng, Wencai Yang, Mai-he Li, Ueli Grossniklaus, Hongkun Zheng, Xiaowu Wang

**Affiliations:** 1 Institute of Vegetables and Flowers, Chinese Academy of Agricultural Science, 100081 Beijing, China; 2 College of Horticulture, China Agricultural University, 100193 Beijing, China; 3 Biomarker Technologies Corporation, 101300 Beijing, China; 4 Department of Plant and Microbial Biology, University of Zürich, 8008 Zürich, Switzerland; 5 Shandong Provincial Key Laboratory of Protected Vegetable Molecular Breeding, Shandong Shouguang Vegetable Seed Industry Group Co. Ltd, 262700 Shouguang, Shandong Province China; 6 Forest Dynamics, Swiss Federal Research Institute WSL, 8903 Birmensdorf, Switzerland

**Correction to:Horticulture Research**

10.1038/s41438-018-0071-9, published online 15 August 2018.

Since the publication of this article, the authors have noticed that the total gene models (45,985), tandem arrays (2077), tandem genes (4963), redundancy removed (43,099), syntenic genes (39,858), nonsyntenic genes (3241), genes on chromosomes (45,411), genes on scaffolds (574) of *B. rapa* reference genome v3.0 were mistaken in the article.

For *B. rapa* reference genome v3.0, the total gene models should be 46,250, tandem arrays should be 2317, tandem genes should be 5584, redundancy removed should be 42,983, syntenic genes should be 39,901, nonsyntenic genes should be 3082, genes on chromosomes should be 45,595 and genes on scaffolds should be 655.

We also update the genes on the three subgenomes in Supplementary table s14.

The authors would like to apologize for these errors.

## Supplementary information

Supplementary Table 14

