# Supplementary material for: Improved Brassica rapa reference genome by single-molecule sequencing and chromosome conformation capture technologies
Source: Hortic Res. 2018 Aug 15;5:50. doi: 10.1038/s41438-018-0071-9 (PMC6092429; doi:10.1038/s41438-018-0071-9)
Supplement: Supplementary file 1 — Supplementary_Figures [file 41438_2018_71_MOESM1_ESM.docx]

**Supplementary Figures**


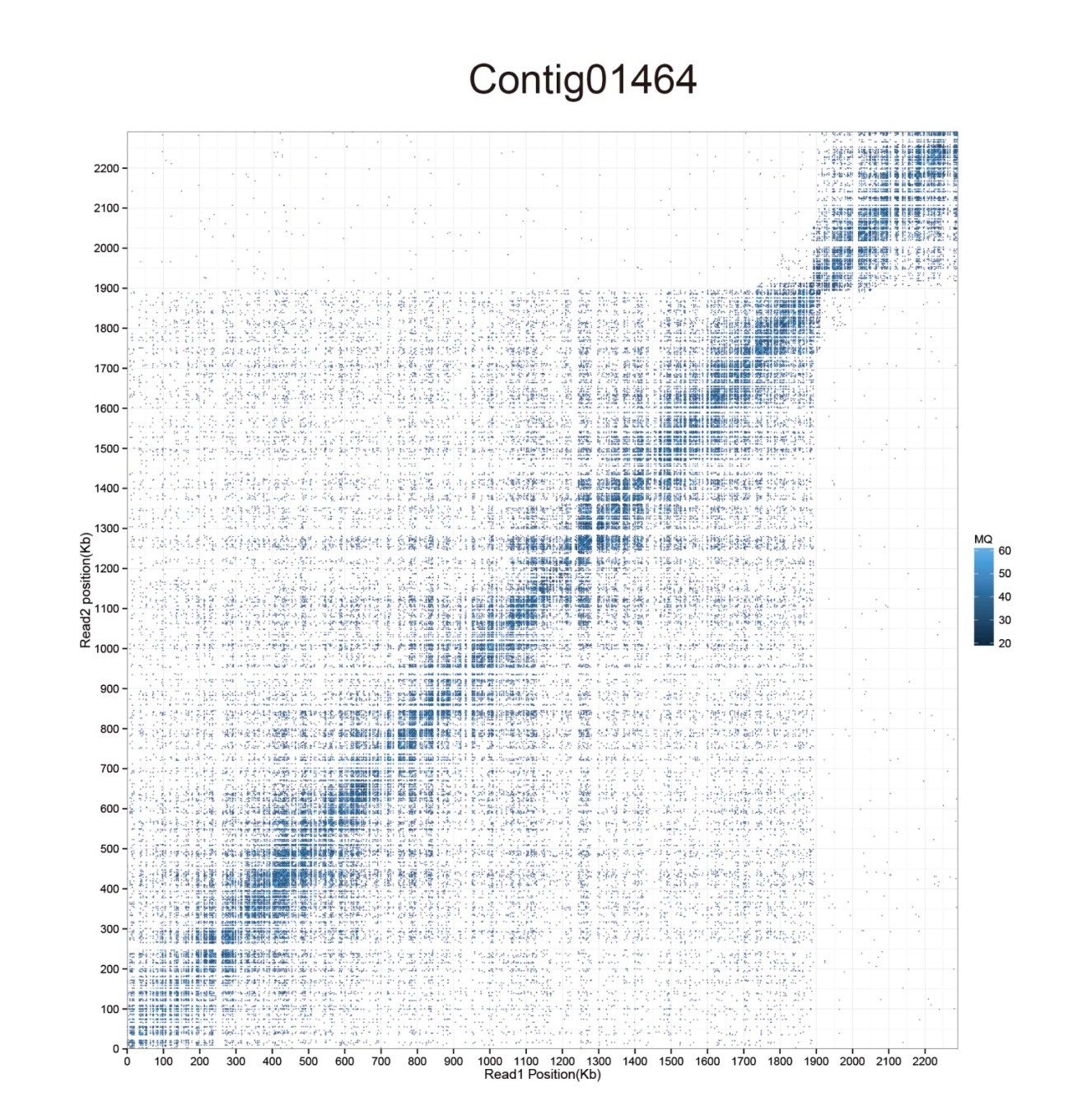


**Supplementary Figure S1.** **An example for the identification of a misassembled contig using Hi-C data.** A misassembly in the PacBio contig01464 was indicated by a sudden absence of read pairs spanning across the region at around 1900 kb. (MQ) Mapping quality.


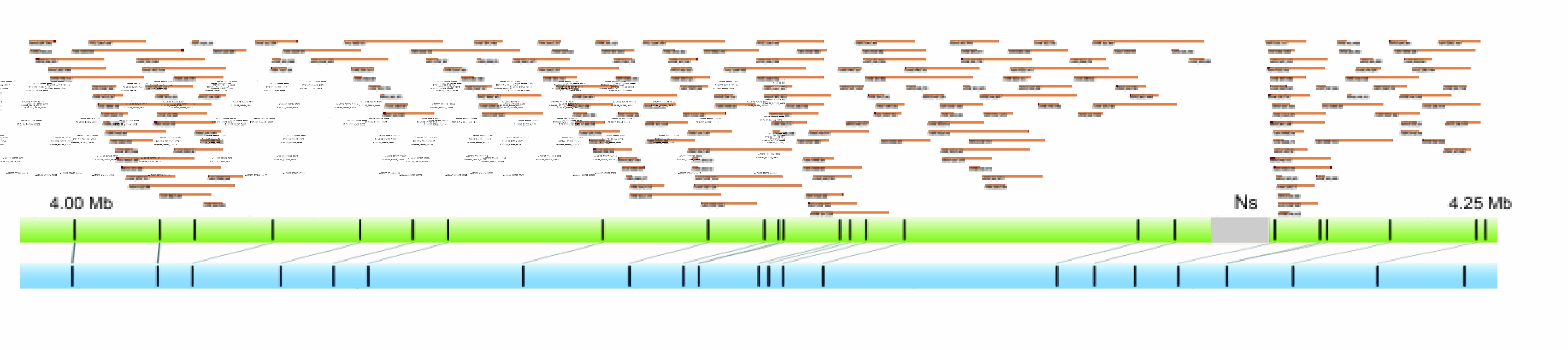


**Supplementary Figure S2.** **Graphical representation of the gap on chromosome A08 of v3.0.** There is a gap at around 4,210 kb which may led to the misassembly that introduced the conflict in the order of markers on linkage group LG08. However, it was fully covered by BioNano maps, supporting the correct assembly of this region on chromosome A08.

**
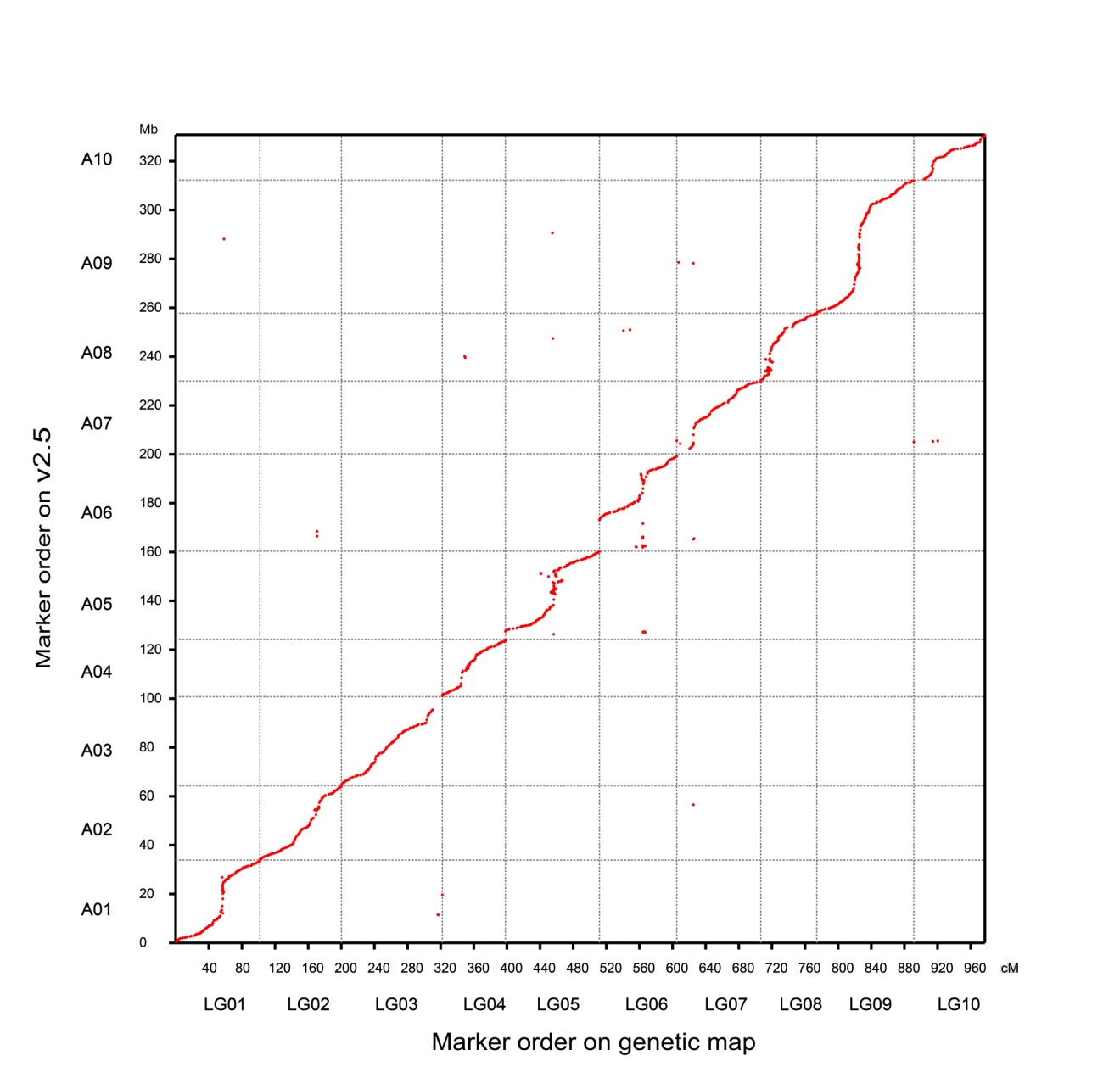
**

**Supplementary Figure S3.** **Integration of physical and genetic maps of *B. rapa* genome v2.5.** The markers of genetic map based on v2.5 are shown on the x-axis; the markers of the physical map of v2.5 are shown on the y-axis.


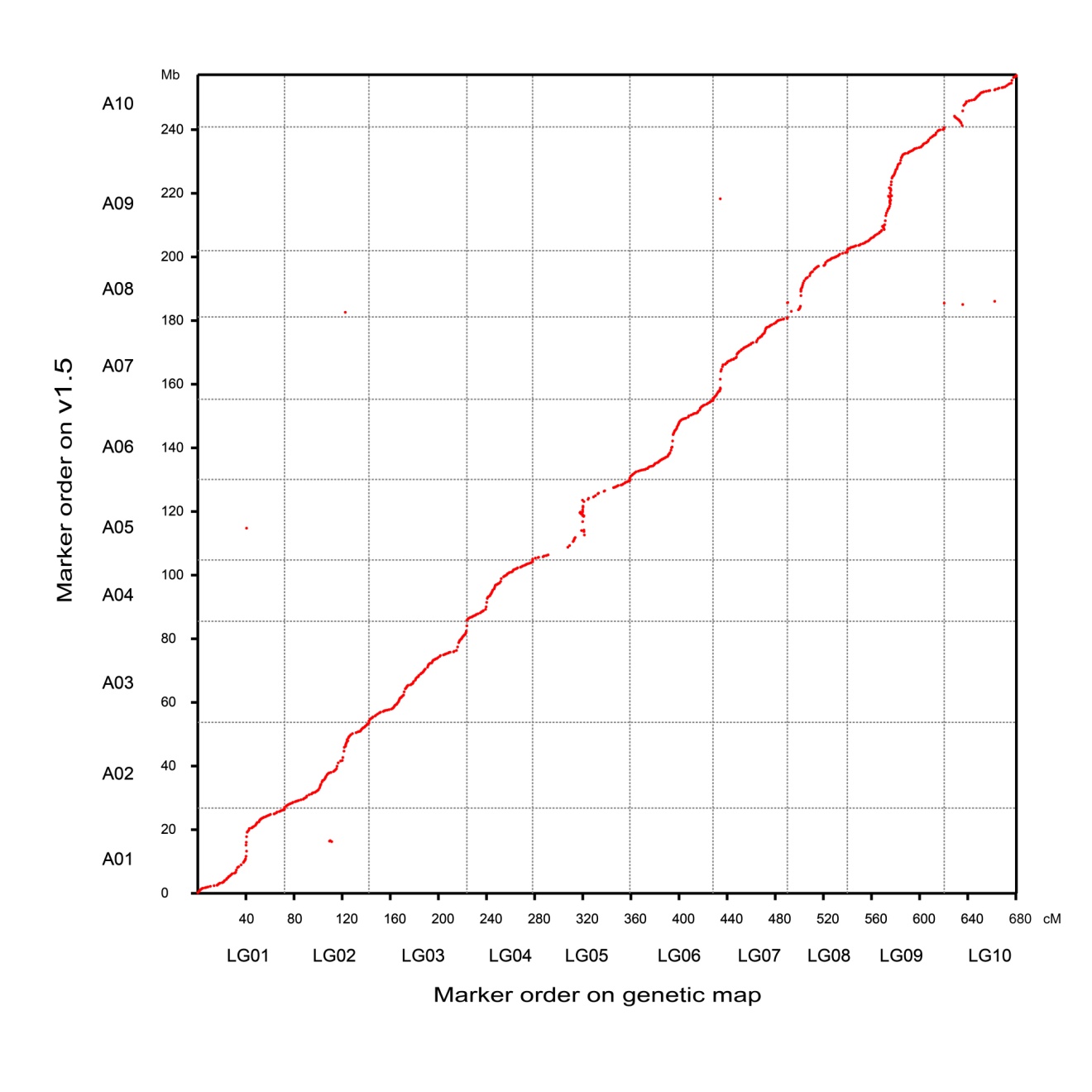


**Supplementary Figure S4.** **Integration of physical and genetic maps of *B. rapa* genome v1.5.** The markers of genetic map based on v1.5 are shown on x-axis; the markers of the physical map of v1.5 are shown on y-axis.


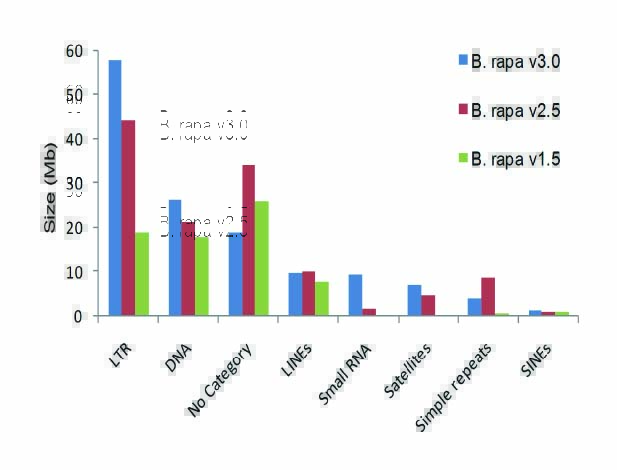


**Supplementary Figure S5.** **Comparisons of TEs and repeats in the three *B. rapa* assemblies, v3.0 (blue), v2.5 (red), and v1.5 (green).**


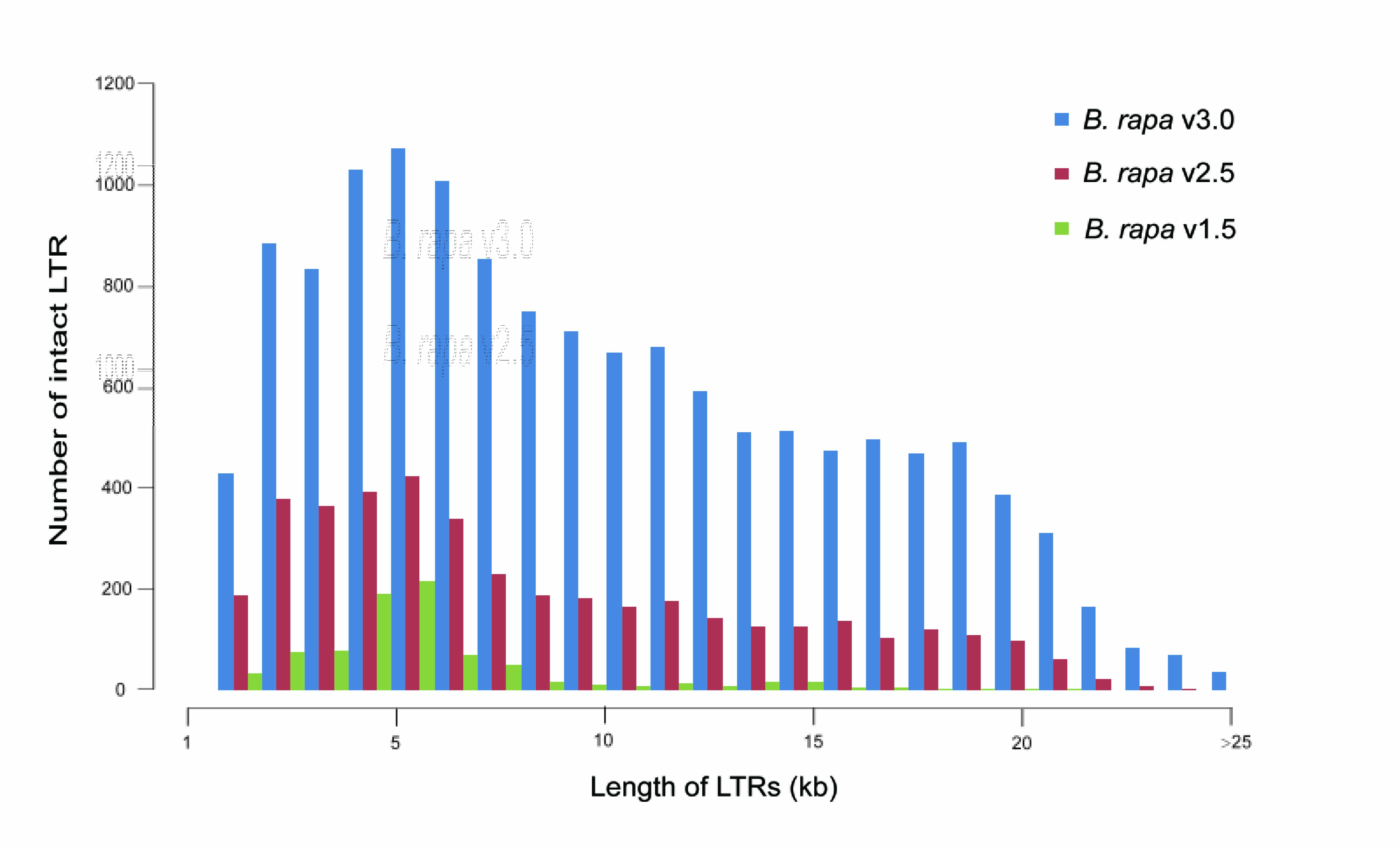


**Supplementary Figure S6.** **The number of intact LTR-RTs with different length (kb) in three *B. rapa* genome assemblies.**


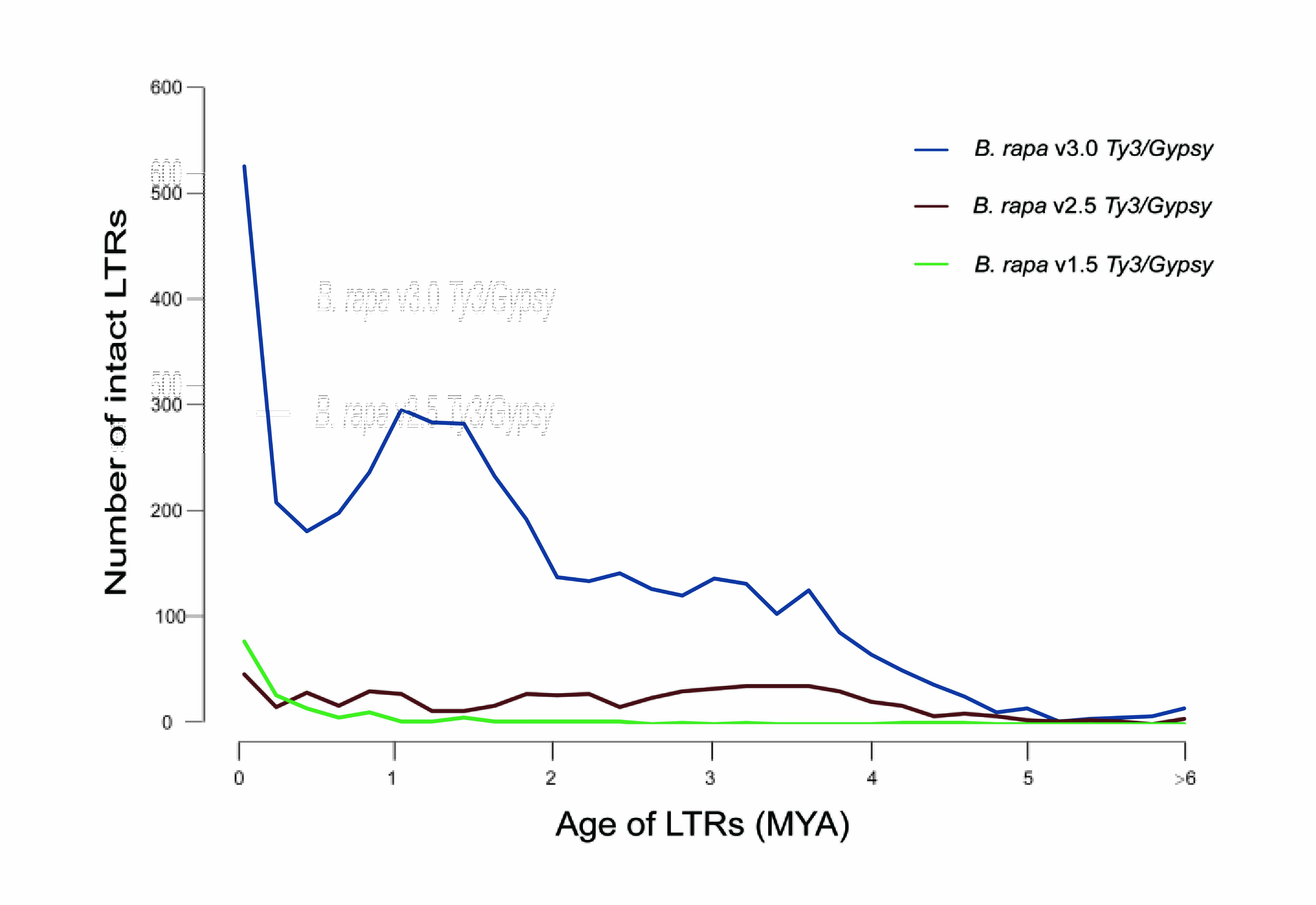


**Supplementary Figure S7.** **The distribution of *Ty3/Gyspy*-like intact LTR-RTs at different insertion times (MYA) in three *B. rapa* genome assemblies.**


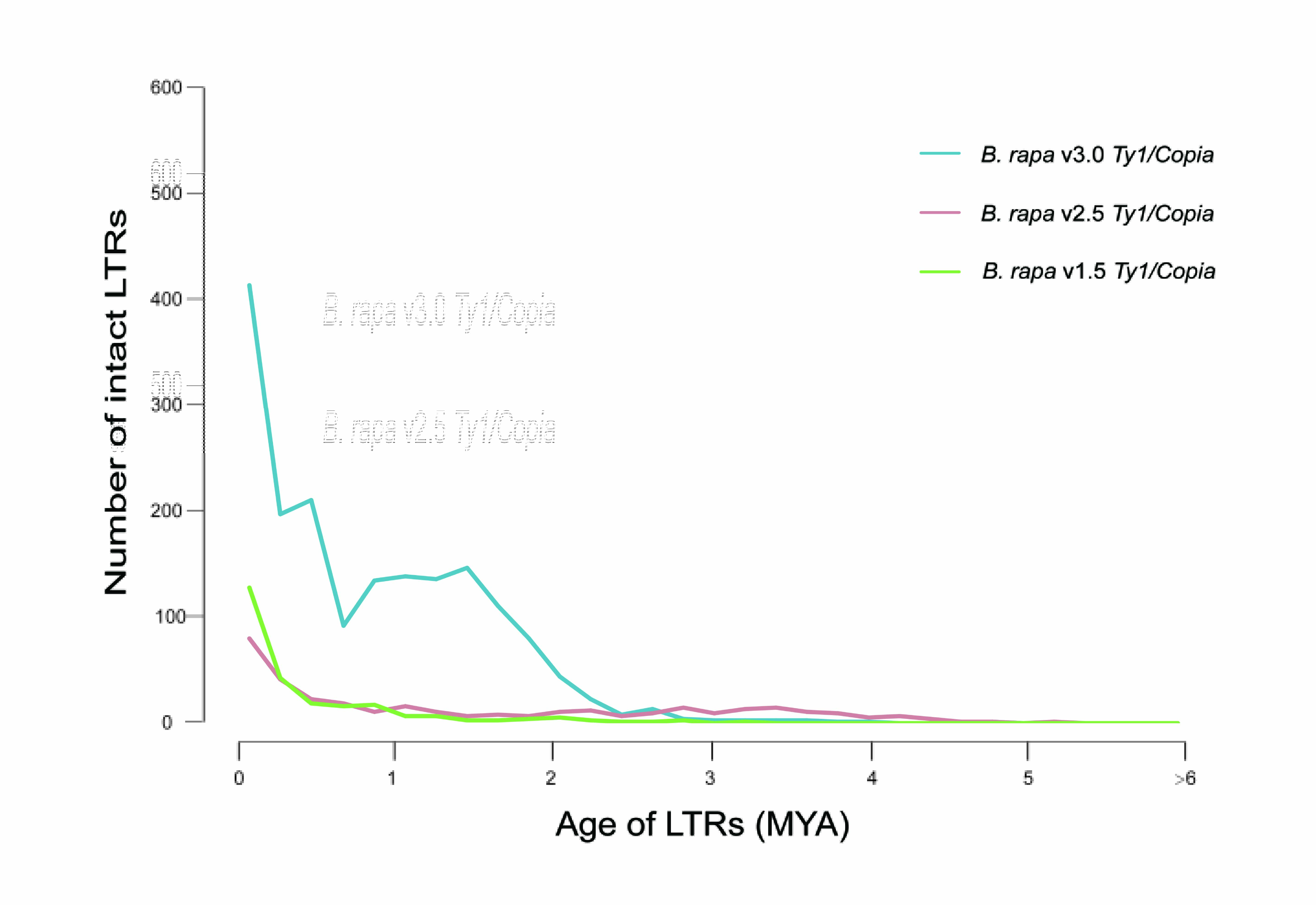


**Supplementary Figure S8.** **The distribution of *Ty1/Copia*-like intact LTR-RTs at different insertion times (MYA) in three *B. rapa* genome assemblies.**


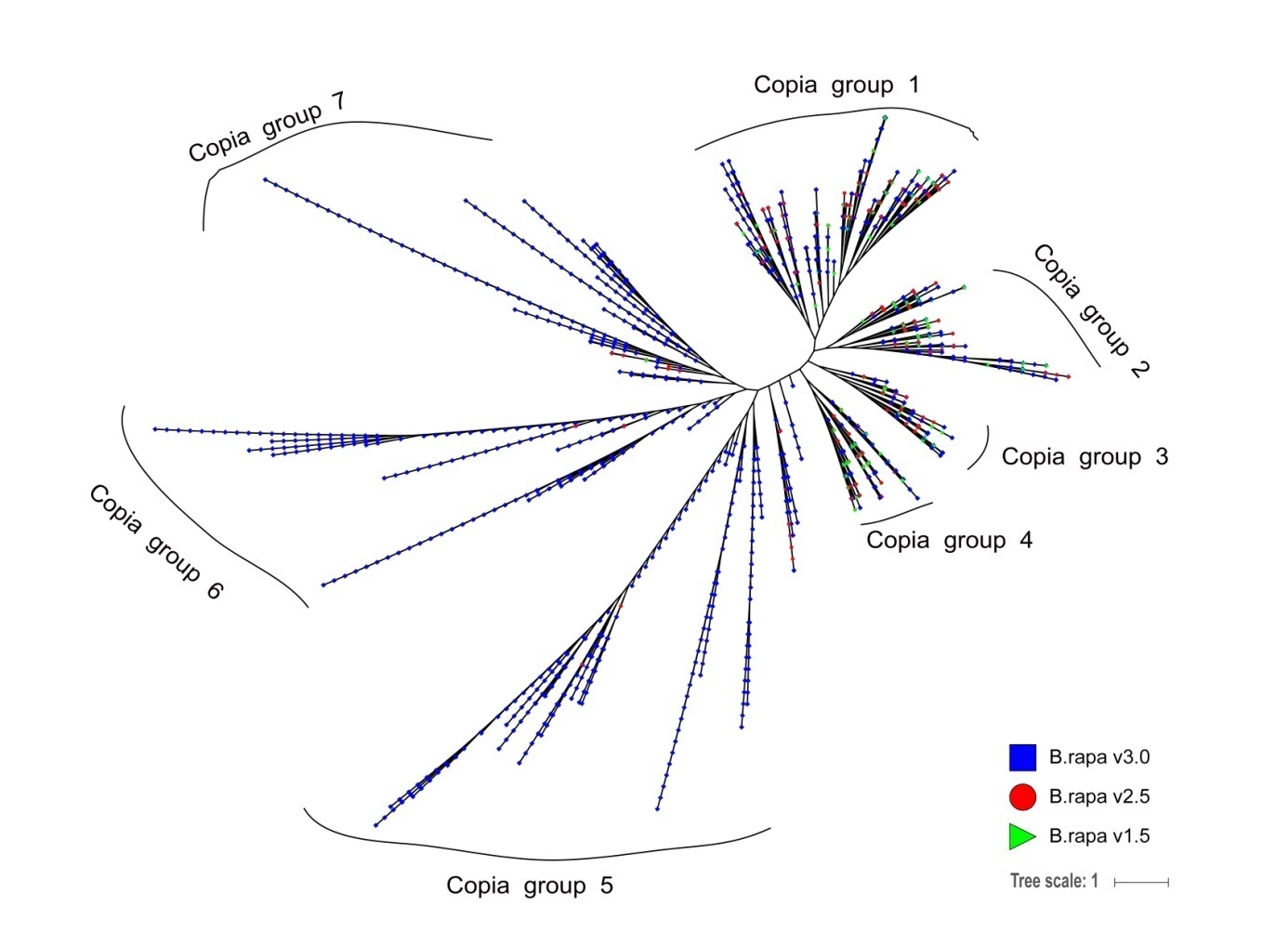


**Supplementary Figure S9. Phylogenetic trees of *Ty1/Copia*-like intact LTR-RTs in the three assemblies of the *B. rapa* genome.** RT amino acid sequences for individual intact LTR-RTs were used to constructed the phylogenetic trees.


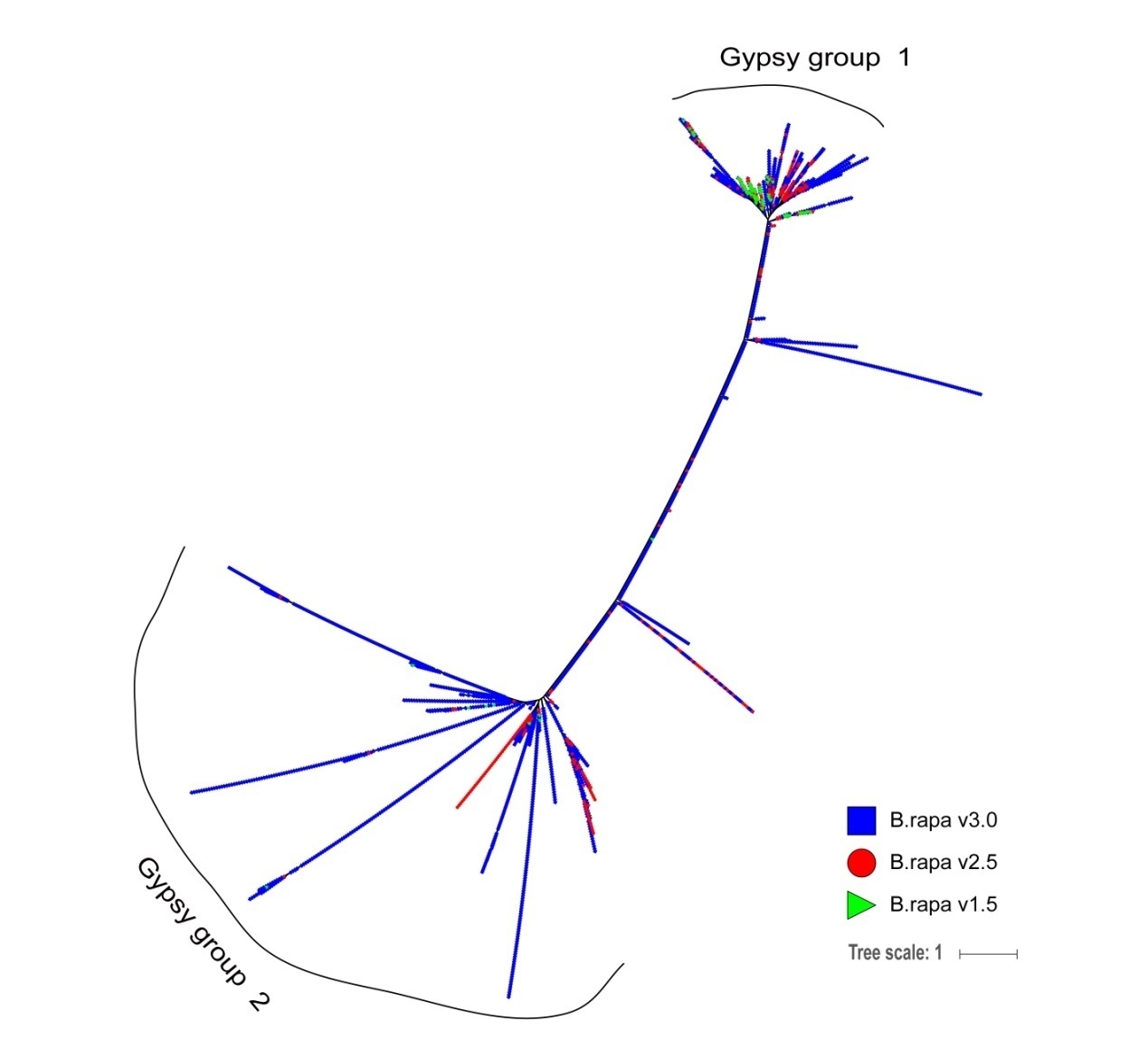


**Supplementary Figure S10. Phylogenetic trees of *Ty3/Gypsy*-like intact LTR-RTs in the three assemblies of the *B. rapa* genome.** RT amino acid sequences for individual intact LTR-RTs were used to constructed the phylogenetic trees.


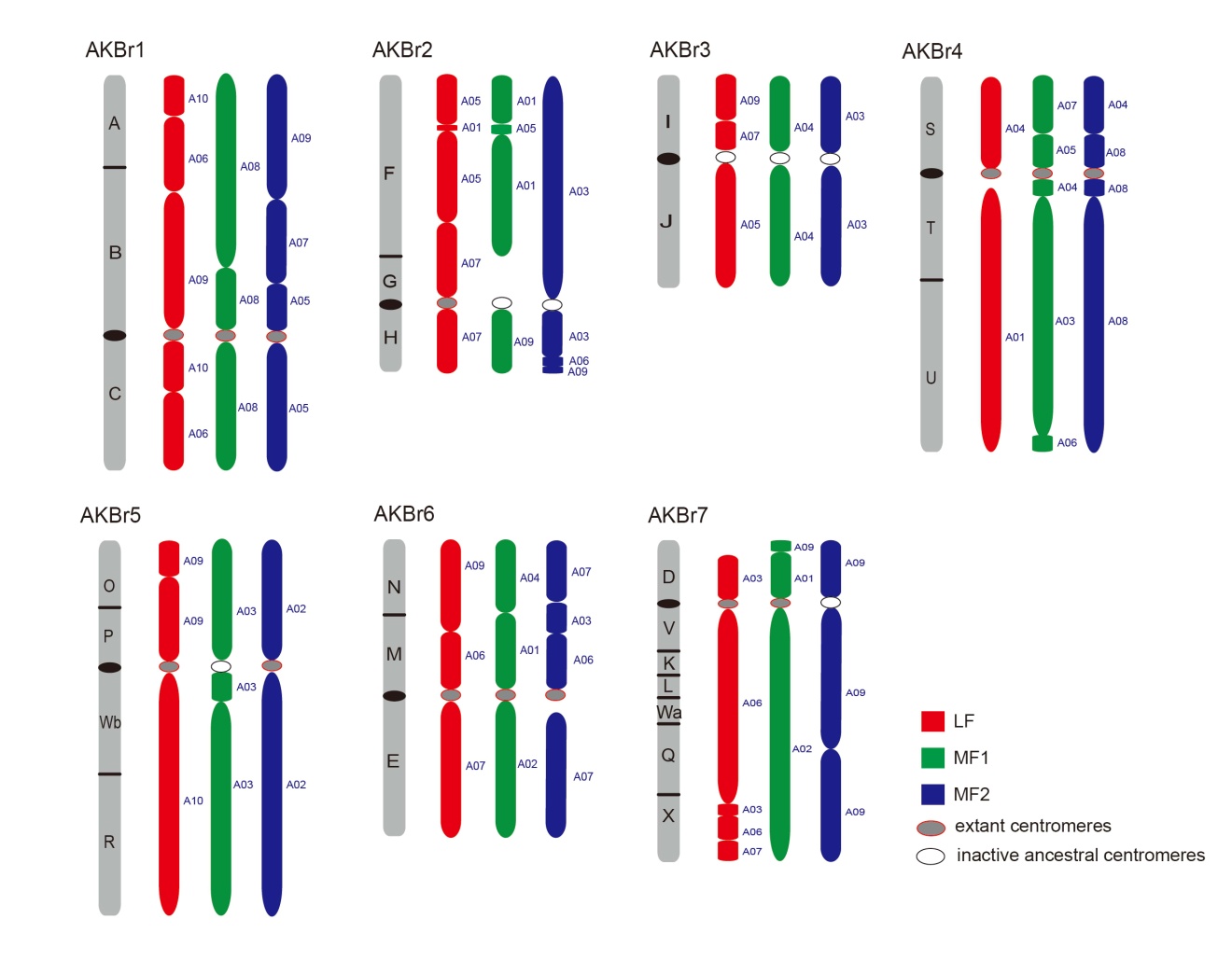


**Supplementary Figure S11.** **Reconstruction of the three ancestral subgenomes in *B. rapa* genome v3.0.**
