# Supplementary material for: Improved Brassica rapa reference genome by single-molecule sequencing and chromosome conformation capture technologies
Source: Hortic Res. 2018 Aug 15;5:50. doi: 10.1038/s41438-018-0071-9 (PMC6092429; doi:10.1038/s41438-018-0071-9)
Supplement: Supplementary file 2 — Supplementary_Tables [file 41438_2018_71_MOESM2_ESM.docx]

**Supplementary Tables**

**Supplementary Table S1.** **Genome size estimation of *B. rapa* by flow cytometry**

| **Biological Replicates** | **Peak value** | **CV (%)** | **Genome size (Mb)** |
| --- | --- | --- | --- |
| *O. sativa* (Nipponbare)/*B. rapa* | 51.92/60.13 | 6.07/3.21 | 450.51 |
| *O. sativa* (Nipponbare)/*B. rapa* | 50.61/59.46 | 4.50/3.96 | 457.02 |
| *O. sativa* (Nipponbare)/*B. rapa* | 47.66/55.02 | 6.25/4.02 | 449.07 |
| *O. sativa* (Nipponbare)/*B. rapa* | 47.57/55.20 | 3.13/3.71 | 451.39 |
| *O. sativa* (Nipponbare)/*B. rapa* | 48.77/58.31 | 2.06/3.36 | 465.09 |
| *O. sativa* (Nipponbare)/*B. rapa* | 48.73/57.44 | 3.45/3.66 | 458.53 |
| Mean |  |  | 455.27 |

**Supplementary Table S2.** **Statistics of BioNano data collection and assembly**

|  | **Number of sequences** | **Total length** | **Coverage** | **N50 size** | **Maximum of sequences (kb)** |
| --- | --- | --- | --- | --- | --- |
| Single molecules  (> 100 kb) | 1,222,404 | 207.7 Gb^a^ | 456 | 170.8 Kb | 2,691 |
| BioNano maps | 286 | 442.9 Mb^b^ | - | 2.37 Mb | 12,543 |

^a^ GB = Giga base pairs

^b^ Mb = Mega base pairs

**Supplementary Table S3.** **Metrics of the different steps performed for genome assembly of the *B. rapa* genome v3.0**

|  | **PacBio assembly** | **Hi-C Corrected** | **Mate-Pairs and BioNano Scaffolding** |
| --- | --- | --- | --- |
| Number of sequences | 1,476 | 1,498 | 1,301 |
| Sequence total size (kb) | 351,064 | 351,064 | 353,141 |
| N50 size (kb) | 1,446 | 1,394 | 4,437 |
| Maximum of sequences (kb) | 9,418 | 9,418 | 20,138 |

**Supplementary Table S4.** **Lists of misassembled contigs and their split information in *B. rapa* genome v3.0**

This table is provided as Microsoft Excel spreadsheet.

**Supplementary Table S5.** **Detailed information on the order and orientation of scaffolds on chromosomes of *B. rapa* genome v3.0**

This table is provided as Microsoft Excel spreadsheet.

**Supplementary Table S6.** **Statistics of the completeness of the three *B. rapa* assemblies by CEGMA**

| **Version** | **Complete** | | **Partial** | |
| --- | --- | --- | --- | --- |
|  | **Protein Num.^a^** | **Completeness(%)^b^** | **Protein Num.^a^** | **Completeness(%)^b^** |
| v3.0 | 247 | 99.60 | 248 | 100 |
| v2.5 | 245 | 98.79 | 247 | 99.60 |
| v1.5 | 246 | 99.19 | 247 | 99.60 |

^a^ Protein Num**.**: Number of 248 ultra-conserved core eukaryotic genes (CEGs) present in three *B. rapa* assemblies.

^b^ Completeness (%): Percentage of 248 ultra-conserved CEGs present in three *B. rapa* assemblies.

**Supplementary Table S7.** **Comparisons of statistics of markers on the genetic maps based on three *B. rapa* assemblies**

| **Version** | **Total makers** | **Mapped makers** | **Conflict markers** | | **Percentage**  **(%)^c^** |
| --- | --- | --- | --- | --- | --- |
|  |  |  | **Intra-chromosome^a^** | **Inter-chromosome^b^** |  |
| v3.0 | 892 | 877 | 76 | 0 | 8.67 |
| v2.5 | 1,092 | 969 | 146 | 20 | 15.07 |
| v1.5 | 866 | 799 | 71 | 9 | 10.01 |

^a^ markers on a certain chromosome were mapped on the corresponding linkage group.

^b^ markers on a certain chromosome were mapped on a different linkage group.

^c^ percentage of conflict markers presented in mapped markers on the genetic maps.

**Supplementary Table S8.** **Detailed information of markers on the genetic map based on *B. rapa* genome v3.0**

This table is provided as Microsoft Excel spreadsheet.

**Supplementary Table S9.** **Detailed information of markers on the genetic map based on *B. rapa* genome v2.5**

This table is provided as Microsoft Excel spreadsheet.

**Supplementary Table S10.** **Detailed information of markers on the genetic map based on *B. rapa* genome v1.5**

This table is provided as Microsoft Excel spreadsheet.

**Supplementary Table S11.** **Statistics of the completeness of gene annotation of three *B. rapa* genome assemblies by BUSCO**

| **Types** | **v3.0** | **v2.5** | **v1.5** |
| --- | --- | --- | --- |
| Complete BUSCOs(%) | 96.70 | 96.20 | 96.40 |
| Fragmented BUSCOs(%) | 1.70 | 1.50 | 1.70 |
| Missing BUSCOs(%) | 1.60 | 2.30 | 1.90 |
| Total BUSCOs searched | 1,440 | 1,440 | 1,440 |

**Supplementary Table S12.** **Comparison of the protein sequence homology between *B. rapa* version-specific genes and other *Brassicaceae* species**

| **Species** | **v3.0** | | **v2.5** | | **v1.5** | |
| --- | --- | --- | --- | --- | --- | --- |
|  | **Hits** | **Percent(%)** | **Hits** | **Percent(%)** | **Hits** | **Percent(%)** |
| *A.thaliana* | 1355 | 41.8% | 1850 | 47.2% | 1346 | 51.2% |
| *A.lyrata* | 1242 | 38.3% | 1757 | 44.9% | 1353 | 51.5% |
| *B.oleacea* | 2243 | 69.2% | 2703 | 69.0% | 2092 | 79.6% |
| *C.rubella* | 1331 | 41.1% | 1800 | 46.0% | 1298 | 49.4% |
| *T.parvula* | 1433 | 44.2% | 1979 | 50.5% | 1363 | 51.8% |
| Total | 2295 | 70.8% | 2779 | 71.0% | 2130 | 81.0% |

**Supplementary Table S13.** **Statistics of the TEs and repeats in the *B. rapa* genome v3.0**

| **Subclass** | **Copy Number** | **Length(kb)** | **%Genome** |
| --- | --- | --- | --- |
| LTR | 51,604 | 57,642 | 16.32% |
| LINE | 16,985 | 9,809 | 2.78% |
| SINE | 6,685 | 1,147 | 0.32% |
| DNA-TE | 71,148 | 26,346 | 7.46% |
| Small RNA | 3,816 | 9,298 | 2.63% |
| Satellite | 2,666 | 7,100 | 2.01% |
| Simple repeat | 3,805 | 3,894 | 1.10% |
| No category | 78,974 | 18,718 | 5.30% |
| Total | 235,683 | 133,952 | 37.93% |

**Supplementary Table S14. Detailed subgenomes information of *B. rapa* genome v3.0**

This table is provided as Microsoft Excel spreadsheet.

**Supplementary Table S15.** **Genomic blocks of the ancestral crucifer karyotype (ACK) in *B. rapa* genome v3.0**

This table is provided as Microsoft Excel spreadsheet.

**Supplementary Table S16.** **Centromere-specific sequences detected in the *B. rapa* genome v3.0**

This table is provided as Microsoft Excel spreadsheet.

**Supplementary Table S17.** **Statistics of signals from centromere-specific sequences and genes in the ten centromeres in *B. rapa* genome v3.0 and v1.5**

| **Centromere** | **v3.0** | | **v1.5** | |
| --- | --- | --- | --- | --- |
|  | **Signals** | **Genes** | **Signals** | **Genes** |
| CentA01 | 1,699 | 104 | 92 | 181 |
| CentA02 | 3,665 | 60 | 26 | 116 |
| CentA03 | 2,624 | 146 | 7 | 8 |
| CentA04 | 3,740 | 33 | 9 | 22 |
| CentA05 | 943 | 63 | 326 | 79 |
| CentA06 | 1,268 | 91 | 151 | 62 |
| CentA07 | 1,643 | 84 | 202 | 27 |
| CentA08 | 1,538 | 93 | 26 | 30 |
| CentA09 | 1,265 | 347 | 28 | 115 |
| CentA10 | 9,456 | 167 | 53 | 100 |
| Total | 27,841 | 1,188 | 920 | 740 |

**Supplementary Table S18.** Summary of the functional annotation in *B. rapa* genome v3.0

| **Annotation database** | **Annotated number** | **Percentage (%)** |
| --- | --- | --- |
| GO | 21,770 | 47.34 |
| Swissprot | 33,811 | 73.53 |
| KEGG | 41,256 | 89.72 |
| TrEMBL | 44,511 | 96.79 |
| Total | 44,539 | 96.86 |

**Supplementary Table S19.** **Summary of non-coding RNAs in three *B. rapa* assemblies**

| **Type** | **v3.0** | **v2.5** | **v1.5** |
| --- | --- | --- | --- |
| miRNA | 1,224 | 1,295 | 680 |
| tRNA | 1,281 | 1,391 | 1,180 |
| rRNA | 2,865 | 1,730 | 153 |
| snRNA | 3,738 | 3,511 | 2,999 |

**Supplementary Table S20. Summary of intact LTR-RTS in three *B. rapa* assemblies.**

| **Version** | **Type** | **Copy** | **Total Length(bp)** | **Average Length(bp)** |
| --- | --- | --- | --- | --- |
| v3.0 | *Ty3/Gypsy* | 4,179 | 56,198,369 | 13,447.80 |
|  | *Ty1/Copia* | 1,778 | 22,149,864 | 12,457.74 |
| v2.5 | *Ty3/Gypsy* | 632 | 7,683,234 | 12,157.02 |
|  | *Ty1/Copia* | 353 | 2,341,893 | 6,634.26 |
| v1.5 | *Ty3/Gypsy* | 162 | 1,244,519 | 7,497.10 |
|  | *Ty1/Copia* | 260 | 1,443,847 | 5,553.25 |

**Supplementary Table S21. Statistics of copy number of *Ty1/Copia* like intact LTR-RTs in three *B. rapa* genomes**

| Type | v3.0 | v2.5 | v1.5 |
| --- | --- | --- | --- |
| Copia_group1 | 835 | 314 | 143 |
| Copia_group2 | 113 | 63 | 54 |
| Copia_group3 | 46 | 43 | 37 |
| Copia_group4 | 55 | 44 | 48 |
| Copia_group5 | 213 | 3 | 0 |
| Copia_group6 | 179 | 2 | 0 |
| Copia_group7 | 185 | 8 | 4 |
| Copia_others | 43 | 8 | 0 |

**Supplementary Table S22. Statistics of copy number of *Ty3/Gypsy* like intact LTR-RTs in three *B. rapa* genomes**

| Type | v3.0 | v2.5 | v1.5 |
| --- | --- | --- | --- |
| Gypsy_group1 | 1480 | 177 | 9 |
| Gypsy_group2 | 1047 | 131 | 8 |
| Gypsy_others | 421 | 47 | 1 |
